# Genetic heterogeneity revealed by sequence analysis of *Mycobacterium tuberculosis* isolates from extra-pulmonary tuberculosis patients

**DOI:** 10.1186/1471-2164-14-404

**Published:** 2013-06-17

**Authors:** Sarbashis Das, Tanmoy Roychowdhury, Parameet Kumar, Anil Kumar, Priya Kalra, Jitendra Singh, Sarman Singh, HK Prasad, Alok Bhattacharya

**Affiliations:** 1 School of Computational and Integrative Sciences, Jawaharlal Nehru University, New Delhi, India; 2 Department of Biotechnology, All India Institute of Medical Sciences, New Delhi, India; 3 Division of Clinical Microbiology and Molecular Medicine, Department of Laboratory Medicine, All India Institute of Medical Sciences, New Delhi, India; 4 School of Life Sciences, Jawaharlal Nehru University, New Delhi, India

**Keywords:** Extra-pulmonary Tuberculosis, Next-generation Sequencing, Genetic Heterogeneity, Single Nucleotide Variations, Insertion Elements, Phylogeny, Spoligotyping

## Abstract

**Background:**

Tuberculosis remains a major public health problem. Clinical tuberculosis manifests often as pulmonary and occasionally as extra-pulmonary tuberculosis. The emergence of drug resistant *tubercle* bacilli and its association with HIV is a formidable challenge to curb the spread of tuberculosis. There have been concerted efforts by whole genome sequencing and bioinformatics analysis to identify genomic patterns and to establish a relationship between the genotype of the organism and clinical manifestation of tuberculosis. Extra-pulmonary TB constitutes 15–20 percent of the total clinical cases of tuberculosis reported among immunocompetent patients, whereas among HIV patients the incidence is more than 50 percent. Genomic analysis of *M. tuberculosis* isolates from extra pulmonary patients has not been explored.

**Results:**

The genomic DNA of 5 extra-pulmonary clinical isolates of *M. tuberculosis* derived from cerebrospinal fluid, lymph node fine needle aspirates (FNAC) / biopsies, were sequenced. Next generation sequencing approach (NGS) was employed to identify Single Nucleotide Variations (SNVs) and computational methods used to predict their consequence on functional genes. Analysis of distribution of SNVs led to the finding that there are mixed genotypes in patient isolates and that many SNVs are likely to influence either gene function or their expression. Phylogenetic relationship between the isolates correlated with the origin of the isolates. In addition, insertion sites of IS elements were identified and their distribution revealed a variation in number and position of the element in the 5 extra-pulmonary isolates compared to the reference *M. tuberculosis* H37Rv strain.

**Conclusions:**

The results suggest that NGS sequencing is able to identify small variations in genomes of *M. tuberculosis* isolates including changes in IS element insertion sites. Moreover, variations in isolates of *M. tuberculosis* from non-pulmonary sites were documented. The analysis of our results indicates genomic heterogeneity in the clinical isolates.

## Background

Tuberculosis is a public health challenge. It is estimated that one third of the human population harbor *M. tuberculosis,* however approximately only 20% of these infected individuals go on to develop clinical tuberculosis [1]. The infection with *M. tuberculosis* usually results in pulmonary tuberculosis but it can also manifest in extra-pulmonary sites, such as tuberculous meningitis, endometritis, lymphadenitis, pleuritis, *etc.*[2,3]. In India, around 15–20 percent cases of tuberculosis among immuno-competent adults have been reported to occur at extra-pulmonary sites, whereas among HIV co-infected patients the incidence increases to more than 50% [4]. The classical route of infection is by inhalation of infectious droplets (respiratory route), however, infection can occasionally occur via alternate routes such as skin abrasions and open wounds [5]. Extra-pulmonary tuberculosis has been always recognized as a sequel to primary pulmonary infection [6-8]. How exactly it occurs and its facilitation remains an enigma. The present study has been designed to sequence and analyze extra-pulmonary isolates to identify genomic patterns and features of *M. tuberculosis* isolated from patients with extra-pulmonary tuberculosis.

Genomic variations in *M. tuberculosis* have been studied using a number of different methods, such as spoligotyping and variable-number tandem repeats (VNTRs) [9]*.* These studies have shown variations among different clinical isolates of *M. tuberculosis*. None of these approaches gives a complete picture of variations at the whole genome level, as each of the methods have their limitations. For example, the spoligotyping pattern would be determined by the strain(s) present in the sample under investigation, and it would be difficult to determine if the observed pattern is due to a dominant strain or a collective pattern of all the strains present in the sample Therefore, it is not reliable to use spoligotyping to establish mixed infection. On the other hand, MIRU-VNTR has been considered useful for detecting mixed infection since it’s based on allelic variation. However, it is possible to have different genotypes with the same VNTR pattern, particularly in case of closely related isolates. Efficacy of DNA extraction used in these assays can be potentially hampered by the presence of clumps / aggregation of mycobacteria in clinical samples / cultures.

The genome of *M. tuberculosis* strain H37Rv was sequenced 15 years ago using the standard approach pioneered by Cole *et al*[10]. The analysis of the assembled sequences suggested that the genome size is 4.4 Mb. encoding about 4000 genes [10]. The genome analysis also showed that there are a number of repeat families, particularly PPE and PGRS family of genes. These repeat families of proteins may have a role in pathogenesis. Subsequently, a number of different species and isolates of the *M. tuberculosis* complex have been sequenced. On comparative analysis with other mycobacterial species the MTB complex clustered separately showing a high degree of sequence identity among this group of mycobacterial species [11]. Different isolates showed polymorphisms at the level of single nucleotides, number of repeats at a given loci, indels and synteny [12]. Attempts have been made to map polymorphisms that are correlated with some of the phenotypes, such as drug resistance [13]. Though some correlations have been found a clear cut casual relationship has not been established so far. With the introduction of next generation sequencing, genome sequences of several isolates have become available and now it is possible to identify genetic markers for specific phenotype. It has also become possible to identify evolving patterns in genomes. For example, “hotspot” and “coldspot” regions have been identified using statistical methods and sequence information from large number of isolates [14]. However, most of the genome data available are from *M. tuberculosis* isolates derived from pulmonary tuberculosis patients. Therefore it is relevant that isolates from extra pulmonary isolates derived from tuberculosis patients should also be analyzed at the genome level.

## Results and discussion

### Sequencing of *M. tuberculosis* isolates from extra-pulmonary tuberculosis patients

The details of the patient derived samples and the *M. tuberculosis* clinical isolates used in the study are given in Table 1 and the techniques used for obtaining these isolates have been described in “Methods”. These five isolates were subjected to spoligotyping and the results were compared to the shared-types (ST) and lineages/sub-lineages described in SpolDB4 (http://www.pasteur-guadeloupe.fr/tb/bd_myco.html[15]). Spoligotyping pattern revealed that of the five isolates, F85 and AC544 could be assigned to the Beijing clade and T1 family respectively. However, the remaining three isolates (AC74, LN8 & F99) did not match with any of the previously described lineage of *M. tuberculosis* based on spoligotyping, hence have been categorized as undefined, (Table 2.).

**Table 1:** Description of clinical isolates used in the study and basic statistics of the sequencing data

**Properties**	**AC74**	**AC544**	**F85**	**F99**	**LN8**
Isolation sites	CSF^a^	CSF^a^	FNAC^b^	FNAC^b^	Lymph node from biopsy
Total reads	3611330*2	3045593*2	13609758*2	7100266*2	3072728*2
Average read length	72	72	72	72	72
Total reads aligned after filtering	5572900	4656246	21378146	11116118	4672838
Total reference length	4411532	4411532	4411532	4411532	4411532
% Total reference covered	88.42	91.51	90.17	81.87	86.36
% Reads aligned with reference	96.32	83.78	95.81	94.82	96.72
Optimized average read depth	72.97	53.03	287.71	148.08	60.41

^a^ Cerebro-spinal fluid.

^b^Lymph node using Fine-Needle Aspiration Cytology.

**Table 2:** Spoligotype patterns of the five extra-pulmonary clinical isolates

**S.no.**	**Lab. no.**	**Binary code**	**Octal code**	**ST**	**Lineage**
1	LN 8	□□□■■■□■■■■■■■■■■■■■■■■■■■■■□□□□■□■■□□□■■■■	73777777413071	Unidentified	Unidentified
2	AC 74	■□□□□□□■■■■■■■■■■■■■□□□■■■■■□■■■□□□□■■■■■■■	403777617560771	Unidentified	Unidentified
3	AC 544	■■■■■■■■■■■■■■■■■■■■■■■■■■■■■■■■□□□□■■■■■■■	777777777760771	53	T1
4	F 85	□□□□□□□□□□□□□□□□□□□□□□□□□□□□□□□□□□■■■■■■■■■	3771	1	BEIJING
5	F99	■■■■■■■■■■■■■■■■■■■■■■■■■□■□□□□■□□□□■■■■■■■	777777775020771	Unidentified	Unidentified

DNA was extracted from the isolates as described before [16] and subjected to nucleotide sequencing using NGS technology (IlluminaGA-IIx). Gross statistics derived from sequence data are also shown in Table 1. In general the number of short reads was more than 3 million with an average length of 72 nucleotides for each isolate. We have used the complete genome sequence of *M. tuberculosis* H37Rv strain (NC_000962.2) as the reference sequence. The short reads were aligned to the reference genome as described in “Methods”. While genome coverage varied from 82% to about 92% for different isolates, unaligned reads were between 3 to 16% suggesting that the quality of DNA preparations from these isolates of good quality.

### Sequence annotation and isolate comparison

*M. tuberculosis* H37Rv genome has been reported to encode 3988 protein encoding genes [10]. Nearly 84% of these genes displayed more than 90% coverage based on our alignments in all strains except F99 (54% coverage). Genes with less than 10% coverage and/or read depth below 10 were considered as “missing genes” (see Additional file 1: Table S1). The total number of predicted missing genes in all the five isolates varied from 17 (AC544) to 74 (F99). Some of these genes (total 5) were missing in all the five isolates. Since genomic deletion can result in genes missing in an isolate, further analysis was carried out to identify large deletions in the isolates using Pindel [17], which detects breakpoints for large deletion using paired-end data. Among the missing genes, 62 were predicted by Pindel as those due to genomic deletions. Out of these 62 genomic deletions, 23 and 3 are from F99 and LN8 respectively (see Additional file 1: Table S1). In general a missing gene in one isolate was found in another. LysR family activates divergent transcription of linked target genes or unlinked regulons with diverse functions [18] and it is one such family that was missing in isolate AC74. In yet another example of missing gene is the absence of malonyl CoA-acyl carrier protein transacylase, in all isolates except isolate AC544. This is an essential gene for the transfer of malonyl group from coenzyme-A to acyl carrier protein AcpM, therefore it is required for biosynthesis of cell wall in *M. tuberculosis*[19]. In case of F99 several trans-membrane protein coding genes, such as *Rv2272* and *Rv2273* were missing. Although we have considered repeat regions by realigning short reads with multiple matches, a number of prophage proteins (phiRV2) were not mapped. Prophage proteins are quite often present in and around repeat regions and show polymorphisms with respect to their positions among different isolates. For example, two types of prophage proteins are present in *M. tuberculosis* H37Rv and CDC1551, but absent in *Mycobacterium bovis*[20]. A total of 68 (38%) missing genes are from PE-PGRS gene family.

### Analysis of single nucleotide variations

**Figure 1:**
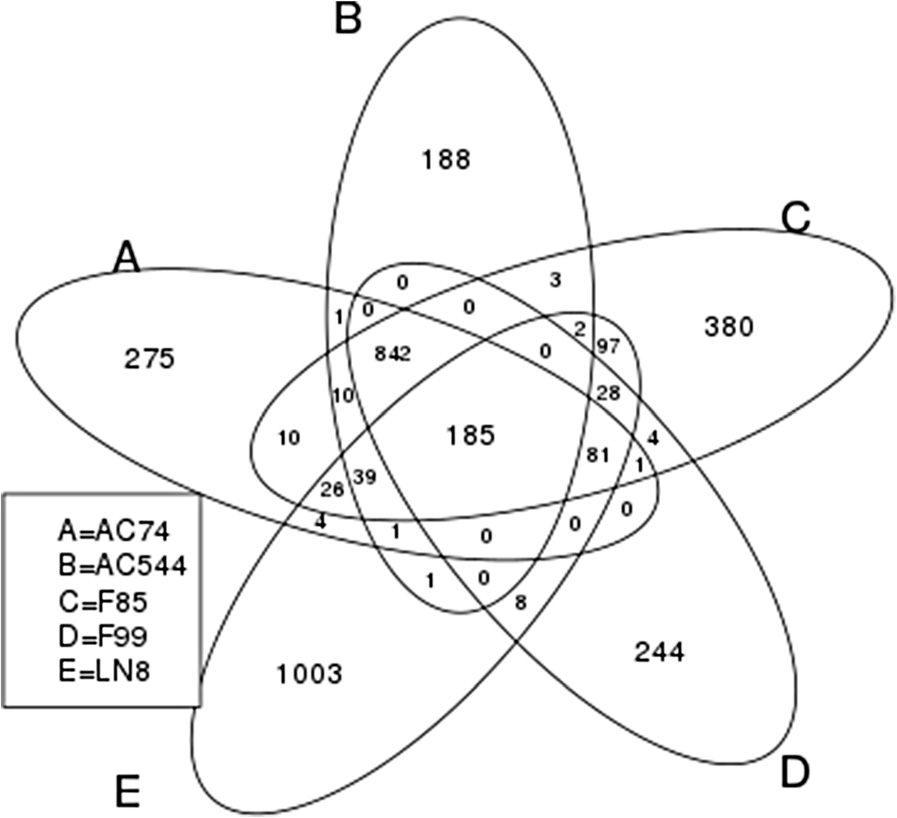
**Common and unique SNVs present in the coding regions of the five isolates.** LN8 has highest number of unique SNVs.

**Figure 2:**
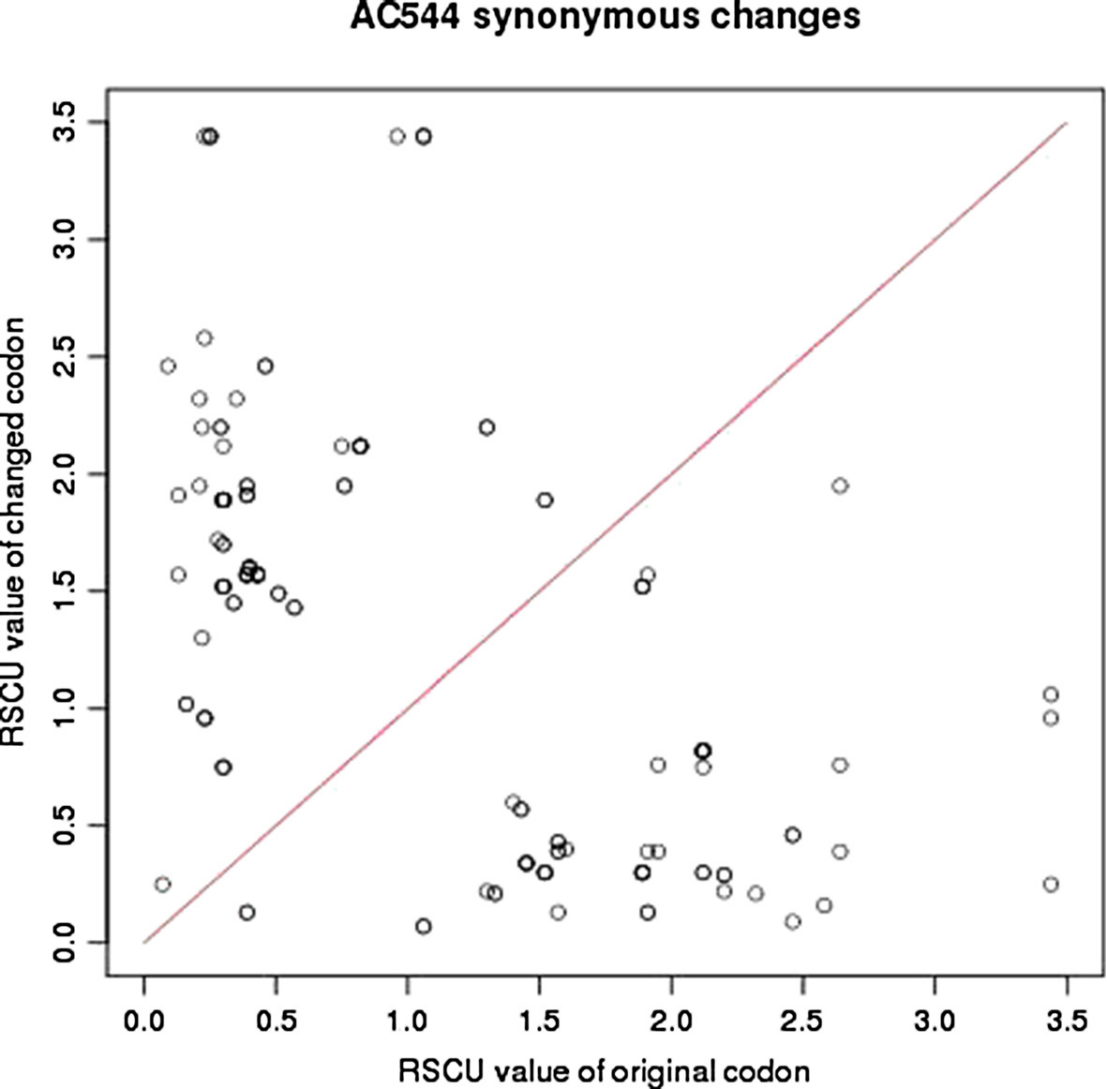
**Plot showing RSCU (Relative Synonymous Codon Usages) of isolate AC544 versus background (*M. tuberculosis *H37Rv).** If RSCU values in an isolate and background are the same then it will lie above the diagonal (red line).

SNVs were classified as major or minor SNVs (see Methods). All major SNVs were grouped either as (a) coding or (b) intergenic depending upon mapping location. To understand the effect of SNVs on the function of respective protein products we further analyzed those that map to coding regions (see Additional file 2: Table S2). A total of 185 SNVs were identified that are common among all isolates in comparison to H37Rv strain (Figure 1). LN8 showed highest number of unique SNVs (not present in other isolates) suggesting that LN8 has diverged much more compared to other isolates. Many SNVs that map to coding regions are likely to cause major changes in the form of gene truncation (by nonsense mutation) and gene elongation (by missense mutation) in different isolates. Some of the SNVs that lead to gene truncation, such as at position 234477 in *Rv0197*, and 1037911 in *Rv0930* (*pstA*) respectively were also observed in a few other isolates of *M. tuberculosis*[21]. Rv0197 protein is a potential oxidoreductase, containing the molybdopterin binding motif. A non-synonymous mutation (arginine to stop) at position 305 was observed in *Rv0930* (phosphate transport integral membrane ABC Transporter, *PstA1* (ABC transporter trans-membrane protein) of isolate LN8, which displays maximum number of non-synonymous mutations that result in stop codons (22) among all five isolates. Beside modification in the gene products, SNVs are likely to affect the level of expression of genes depending upon the usage of altered codons [22]. Relative Synonymous Codon Usage (RSCU) was calculated for all synonymous changes in each strain and the results showed 5–10 fold changes in codon usage for many altered codons as a consequence of SNVs. Some of the examples of genes containing these SNVs are enoyl-CoA hydratase (*Rv0022*), MCE-family proteins (*Rv0591*) and oxidoreductase (*Rv3777*) [23]. RSCU values of original codons and those for isolate AC74 is shown in Figure 2. Details are given in Additional file 3: Table S3. SNVs in the inter-genic regions (specially promoters regions) were also studied (see Additional file 4: Table S10) using the database of promoters from MycoRRdb database as reference [24]. We were able to identify SNV in the promoter of the gene *Rv2779c,* a transcriptional regulator in all isolates except AC544. Though, most of the regulatory regions in MycoRRdb database are computationally predicted motifs, some of these are also mentioned in literature [25-27]. *kstR*2 is one such regulatory motif, shown to be associated with cholesterol utilization in *M.tuberculosis* during infection [26]. One SNV found in the kstR2 motif of 2 genes namely *Rv3560c* gene of F85 isolate and in the *Rv3549c* gene of LN8 isolate, respectively.

### Minor variations

**Figure 3:**
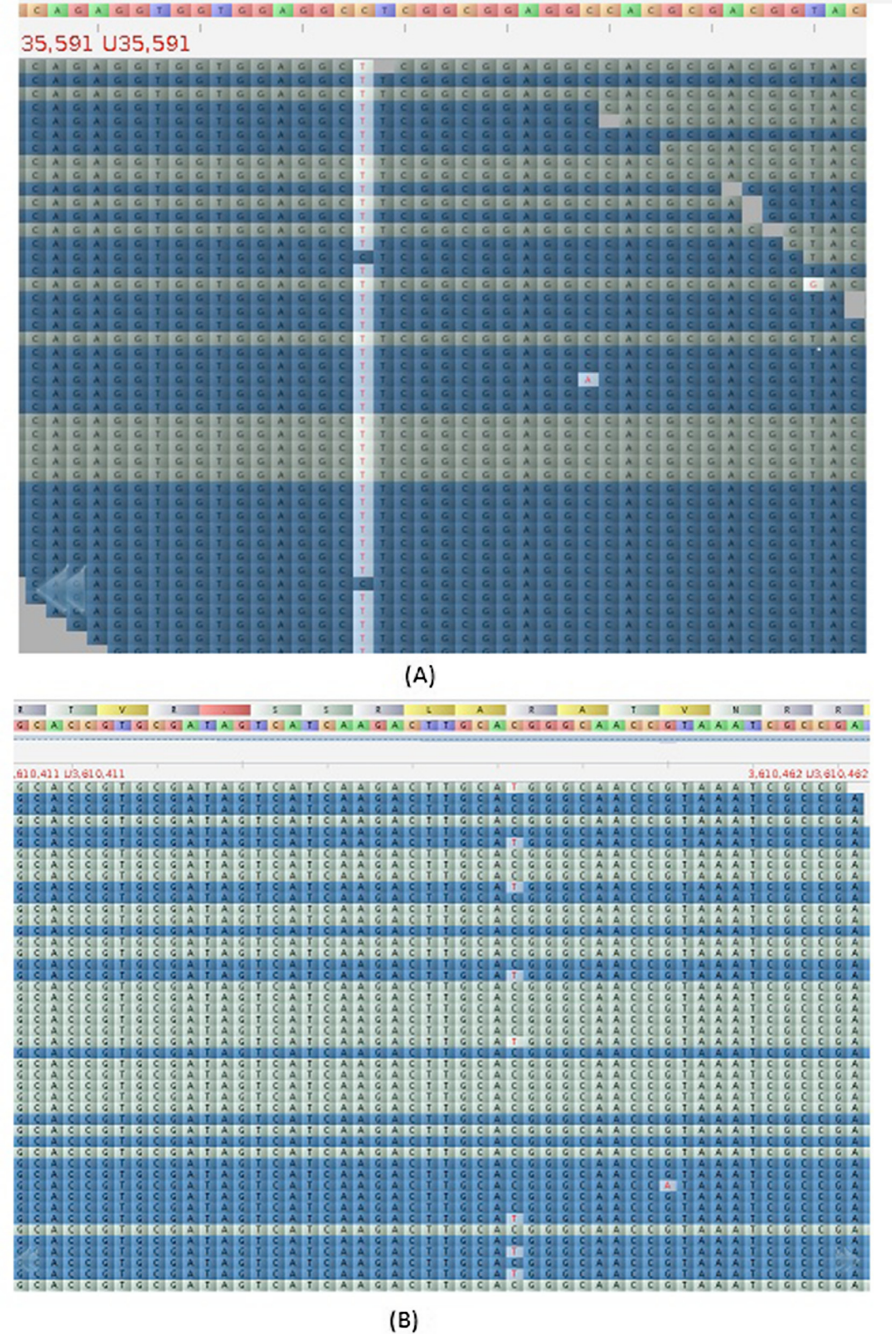
**Examples of major and rare variations are shown from isolates F85.** (**A**) Major variation changes C to T (**B**) rare variation changes C to T.

**Figure 4:**
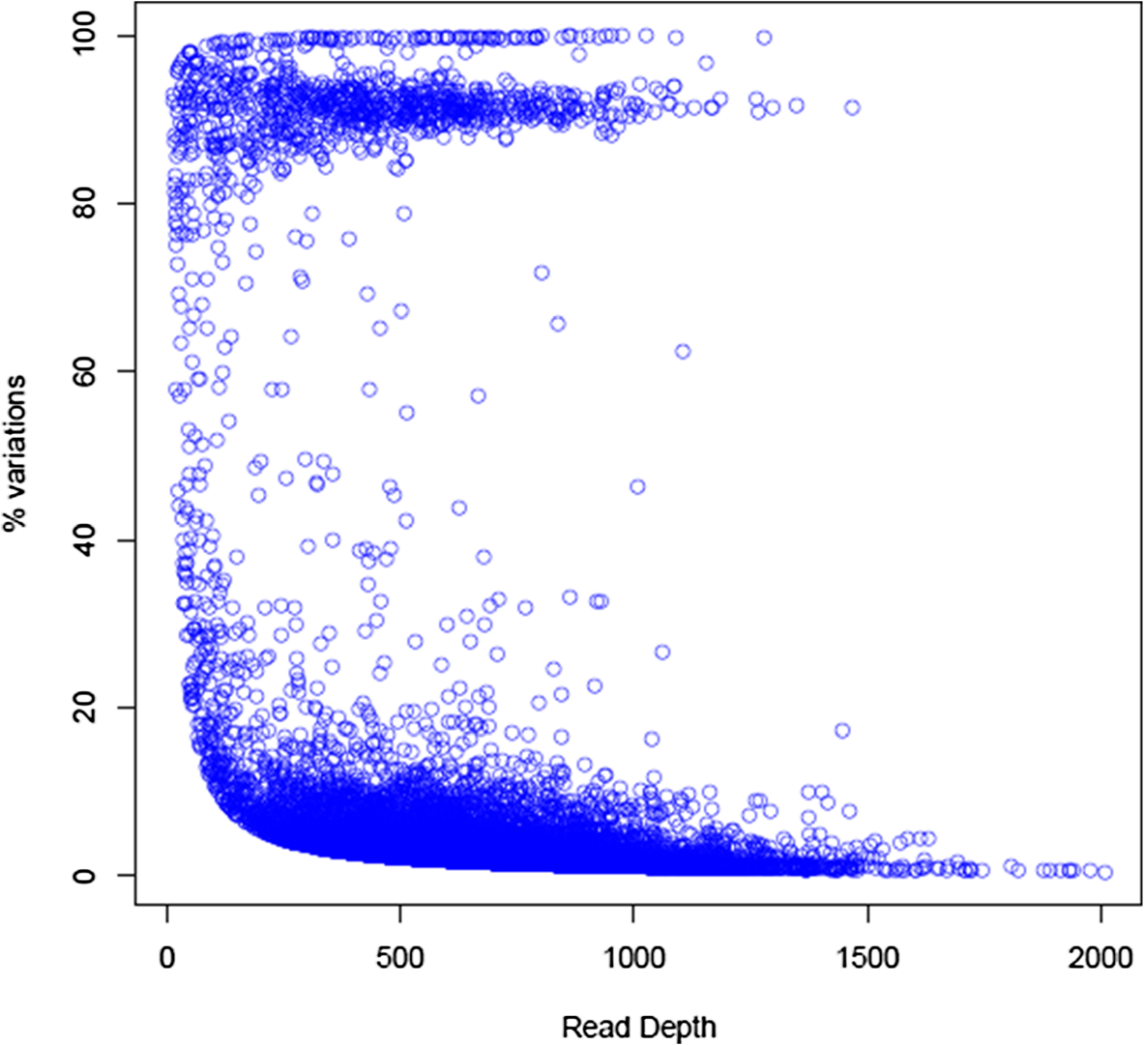
**Plot showing percent variation versus read depth in isolate F85.** Variations below 15% are likely to be due to sequencing error.

**Figure 5:**
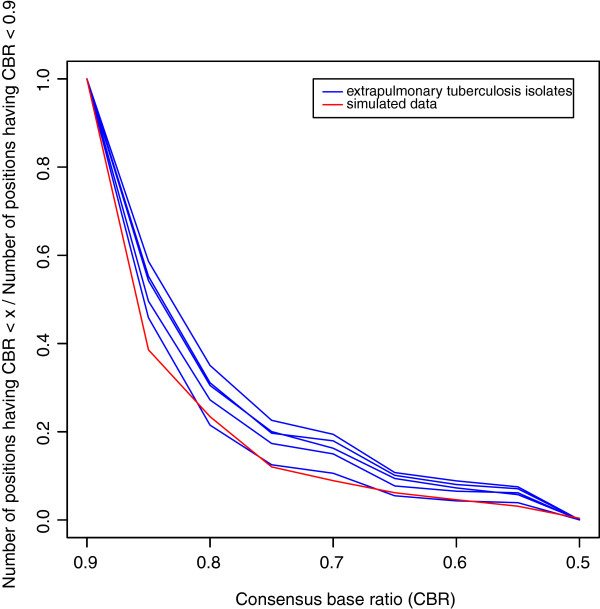
**Distribution of consensus base ratio in the five isolates and in simulated data.** Blue line and red line represent data from isolates and simulation respectively.

We have applied stringent criteria for generation of alignment and also have incorporated paired end reads for identification of genuine SNVs from noise. Typical alignments containing major and minor variants are shown in Figure 3 (A, B). It is unlikely that these minor variants (see Additional file 2: Table S2) are due to sequencing error. When percent variation per nucleotide position was plotted across read-depth, a bimodal distribution was observed (Figure 4). The major component was centered above 95% and the minor one at around 85%. This suggested that the isolates are likely to be a mixture of different genotypes. Our hypothesis was checked by creating simulated data where two strains (*M. tuberculosis* CDC1551 and *M. tuberculosis* H37Rv) were mixed and analysis carried out using our SNV detection system. Variations were identified from simulated data and a comparison with extra-pulmonary isolates is shown in Figure 5. The Blue (represents data from extra-pulmonary isolates) and the red lines (represents simulated data) follow more or less the same path. Different isolates showed mixing of two genotypes and the ratio of the two genotypes varied from 5 to 15%. Mixed genotypes may be due to picking up of more than one colony for expansion and DNA isolation or changes in subsets of cells in patients or during routine culture. Mycobacteria characteristically form clumps, making it difficult to establish clonal populations. We have made all efforts to obtain single colonies. However it is still likely that mixed colonies were picked up for analysis, owing to the inherent ‘clumps’ of mycobacteria. On the other hand it is difficult to reconcile the extent of variations observed, with our current understanding on changes that take place during mycobacterial proliferation.

The likely reason for the origin of mixed genotype could also be due to the fact that patients can be potentially re-infected / super infected by different strains or rapid genomic changes take place in a subset of cells in patients. Recent investigations have suggested that several pulmonary isolates of *M. tuberculosis* display clonal heterogeneity [28,29]. Heterogeneous population does offer selective advantage for survival of the tubercle bacilli within the hostile microenvironment of the host. These alterations may facilitate *in vivo* dissemination / migration of the tubercle bacilli from the pulmonary infectious foci to other organs, as has been speculated to occur in the infected host [30,31].

### Functional classification and enrichment study of genes having major SNVs

**Figure 6:**
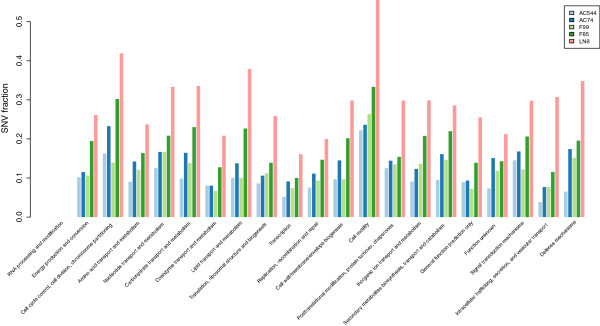
**COG classification of the genes carrying SNVs in the five isolates.** Different COG categories are represented by X-axis. Y-axis represents fraction of SNVs in each category after normalization.

Synonymous and non-synonymous SNVs that map in the coding regions of genes were functionally classified on the basis of COG (Cluster of Orthologous Group) [32]. Functional annotation of uncharacterized proteins was performed according to Doerks *et al*. [33]. Maximum number of SNVs among the isolates was found in genes that belong to COG category N (“Cell motility and secretion”) (Figure 6). *M. tuberculosis* genes in this category are mainly “*ppe”* genes. Polymorphisms in these genes are mostly non-synonymous types. In pathogenic mycobacteria PE/PPE proteins are involved in direct interaction with host immune system [34,35]. Yongjun Li *et al.,* have shown that in *M. avium ppe* genes are associated with growth in macrophages and virulence in mice [36]. Therefore, it is not surprising that these genes undergo high rate of variations compared to housekeeping genes [37]. Biological processes, such as DNA synthesis, transcription and host-pathogen interaction, depend on coordinated functional expression of multiple genes. Therefore gene enrichment analyses were used to identify functionally important genes from SNV data [38]. For these studies, the Databases for Annotation, Visualization and Integrated Discovery tool (DAVID) was used [39,40]. The output from DAVID revealed several gene clusters that are significantly enriched (using Fisher exact p-value). GO terms of these enriched clusters showed that these are essentially membrane bound / trans membrane / two-component system and nucleotide binding proteins. Mutations in these genes would potentially alter biological processes, as these categories of genes are needed by the bacteria for interaction with the environment. Genes that form part of the family of polyketide synthase were also found among the enriched gene sets. The details of enriched clusters including enrichment scores, p-values in different isolates are tabulated in the Additional file 5: Table S4, Additional file 6: Table S5, Additional file 7: Table S6, Additional file 8: Table S7, Additional file 9: Table S8.

### Study of insertion sequence elements (IS elements)

IS elements have been extensively used as a marker for strain identification in *M. tuberculosis* due to high numerical and positional polymorphisms (for a review see [41-44]). IS elements can influence gene expression depending upon the sites of insertion, for example, IS6110 increases the expression of neighboring genes which are involved in virulence [45]. In this study an attempt was made to identify IS elements, particularly IS6110 from short read sequence data of the isolates and to derive a distribution across the genomes. The strategy used has been described in “Methods”. The results are shown in the Additional file 10: Table S9**.**

Total number of IS elements was found to be 56 including 16 copies of IS6110 in H37Rv strain[10]. Our studies revealed that the numbers of copies of IS6110 varied from 5 to 19 in different isolates and their positions differed from that seen in *M. tuberculosis* H37Rv*.*

**Figure 7:**
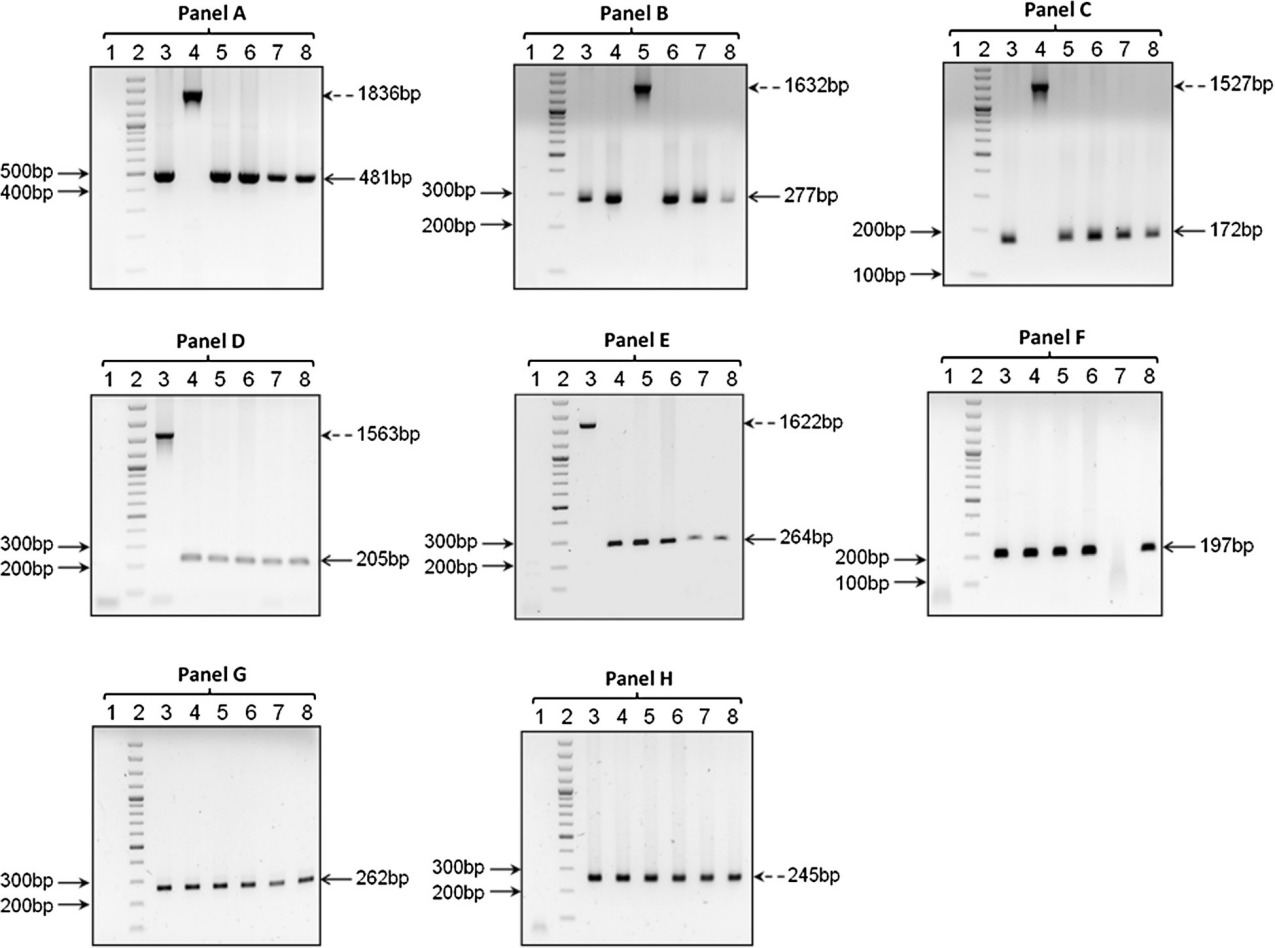
**Analysis of PCR products derived from Primer pairs (Panels A-H) detailed in Table**3**using agarose gel electrophoresis.** Panels **A**, **B** and **C** represent specific coordinates of IS element insertion in the isolates, whereas panels **D** and **E** represent those from where IS element has been translocated in the isolates in comparison to the reference strain H37Rv. Panel **H** (IS6110 internal primers) confirms the presence of IS6110 element in all the strains. Co-ordinates for IS element insertion/deletion and expected amplicon size in the respective strains are detailed in Table 3. Lane 1: Negative control; Lane 2: 100-3000 bp DNA Ladder; Lane 3–8: H37Rv DNA (Lane 3) and Triton extracts of isolates (Lane 4:AC74; Lane 5:AC544; Lane 6:LN8; Lane 7: F85; Lane 8: F99). [Lane 2: DNA Ladder; with IS element; : without IS element].

To validate the predicted translocation or deletion of the IS elements in the 5 extra-pulmonary isolates, PCR amplifications were carried out using primers targeting the regions flanking the predicted coordinates of the IS element. Hence the inclusion of the IS element would result in the generation of an amplicon larger in size compared to the deletion of the IS element in the PCR targeted site. In this regard various primer panels (A-H) were designed (Table 3) to amplify coordinate specified regions of H37Rv and the 5 extra-pulmonary isolates. The presence of IS6110 in the genome of all the isolates was confirmed by PCR (Figure 7, Panel H). Of the 7 pairs of Primer panels used, each targeting a specific positional co-ordinate, PCR products obtained for Panels A, B, D, and E completely matched with the predictions (Figure 7). For example, Panels A and B confirm the presence of the IS element at the predicted co-ordinate 3480373 for the clinical isolate AC74 (Figure 7 Panel A, Lane 4) and at position 1527976 for the clinical isolate AC544 (Figure 7 Panel B, Lane 5), respectively. Similarly, primers described in Panels D and E yielded the expected amplicon of size 205 and 264 bp respectively, as the IS element was predicted to be absent in the 5 clinical isolates, (Figure 7 Panels D, E, Lanes 4–8). Whereas, when the IS element is present, as seen in the reference *M. tuberculosis* H37Rv strain, the expected amplicon size was 1563 bp (co-ordinate 2550014–2551368) and 1622 bp (co-ordinate 1541952–1543306) respectively, (Figure 7 Panels D,E, Lane 3). Panels D and E validate some of the variations in the integration sites of IS element in the 5 extra-pulmonary isolates with respect to *M. tuberculosis* H37Rv. Whereas Panels A, B and C (Figure 7) confirm the insertion of IS element at new locations, in 2 out of 5 isolates with respect to H37Rv strain. Further, it may be noted that the IS element was incorporated into the *Rv3113* gene (putative phosphatase, Figure 7, Panel A) in the clinical isolate AC74. Similarly in the same clinical isolate, another copy of the IS element was found in the *Rv1213* gene (probable glucose-1-phosphate adenylyl-transferase, Figure 7, Panel C) considered important for glycogen synthesis. It is also clear from the data that different isolates may have a different history of movement of these elements. For example, in the clinical isolate AC544, the IS element was observed in *Rv1358* gene (probable transcriptional regulatory protein, Figure 7, Panel B) not observed in other isolates. We could not validate our predictions in a few cases (Figure 7, Panels C, F and G).

**Table 3:** Primer sequences and standardized PCR parameters

**Primer Panels**	**Coordinate of IS element (w.r.t. H37Rv) present in strain**	**Primers flanking the co-ordinate of IS element**	**Standardized annealing temp./ MgCl**_**2 **_**concentration**	**Amplicon size (bp)**				
				**With IS**	**W/o IS**			
A	3480373, + strand, in AC74	5’-CAGCAGGGATGGATTCACC-3’5’-GCGAGTGGGATTCAGAGAG-3’	62°C/1mM	1836	481
B	1527976, + strand, in AC544	5’-GTGGTGCCGTCGTTGTCTC-3’5’-ATGCCCGTAATGTCTGCTGG-3’	60.2°C/1.5mM	1632	277
C	1356494, + strand, in AC741356495, + strand, in F85	5’-TTCGTCTCAATGGGCAACTAC-3’5’-CACCAGGCACTTCGTTATCG-3’	60.2°C/2.5mM	1552	197
D	2550014-2551368, + strand, in H37Rv	5’-CTGCGACTGCGTTGGTAATC-3’5’-ACGGTGGGGAAAGCCTGAAG-3’	61.3°C/1mM	1563	205
E	1541952-1543306, - strand, in H37Rv	5’-AGCAGGAGGAGCGGGACG-3’5’-GGATAACAGGCGCGAACCG-3’	72°C/1mM	1622	264
F	1657017, + strand, in F85	5’-TCCCTACACTCGGTTCATCC-3’5’-ACAGCAGCAGCGCCACGG-3’	61.3°C/1mM	1552	197
G	2229658, - strand, in F99	5’-TGGTGGTCAGGGAAAAGCC-3’5’-CCCTCCCGTAGCAGCCGC-3’	61.3°C/1.5mM	1617	262
H (IS6110^ref^)	Multiple copies present in all	5’-CGTGAGGGCATCGAGGTGGC-3’5’-GCGTAGGCGTCGGTGACAAA-3’	65°C/1mM	245	0

[Ref: Hermans P.W.M., van Soolingen D., Dale J.W. et al., Insertion element IS986 from Mycobacterium tuberculosis: a Useful tool in Diagnosis and Epidemiology of tuberculosis. 1990 J. Clin. Microbiol. 28(9):2051-2058].

### SNV based phylogenetic analysis

**Figure 8:**
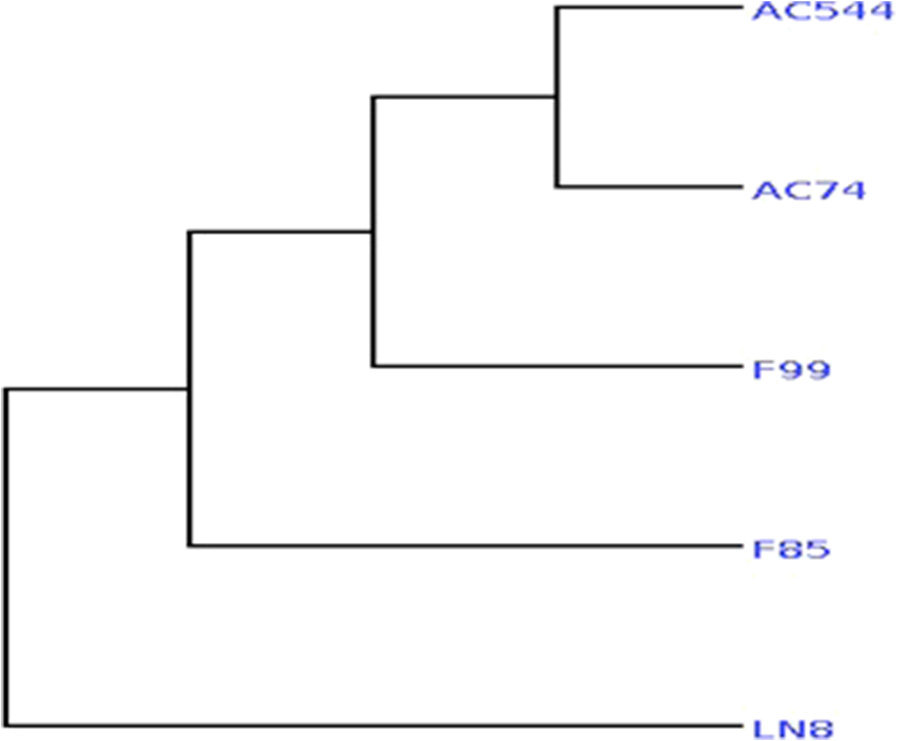
**Phylogenetic relationship.** SNVs were used to derive the tree using “Neighbour Joining” method.

Phylogenetic relationships among isolates were derived from SNVs as described in “Methods”. Distance-based method (“Neighbor Joining”) was used to derive distance estimate between the isolates and branch assignments were validated using bootstrap. The results (Figure 8) showed that isolates AC74 and AC544 were present in the same branch suggesting a common lineage. This is not surprising as both the isolates were obtained from cerebrospinal fluid. F85 and F99 were isolated using FNAC from lymph nodes and these were placed close to CSF branch and separated from LN8, a lymph node derived isolate.

## Conclusions

Our major finding from analysis of NGS data of extra-pulmonary isolates of *M. tuberculosis* is detection of genomic heterogeneity in isolates. The computational approach used by us can identify mixed genotypes even when one of the genotypes is represented at a low level. We have further analyzed the functional significance of SNVs identified by using different approaches, such as COG. NGS data was also utilized to identify IS elements and its insertion into various sites, among the different isolates. Some of these predictions were validated by experiments. Phylogenetic relationship among isolates is consistent with the origin of the isolates.

## Methods

### Mycobacterium strains and genomic DNA isolation

The clinical isolates of *M. tuberculosis* used in this study have been maintained in the TB immunology laboratory, Department of Biotechnology, All India Institute of Medical Sciences (AIIMS), New Delhi, India. These isolates were obtained from clinical samples derived from patients diagnosed with extra-pulmonary tuberculosis. The isolates were obtained from: (1) cerebrospinal fluid (CSF) of patients clinically diagnosed as cases of Tubercular meningitis; (2) from fine needle aspirates / biopsies from patients with lymphadenopathy with discharging sinus / abscess formation, (Table 1). All samples, after due processing, were inoculated on Lowenstein-Jensen (LJ) slants for primary isolation. Separate single colonies were propagated on LJ media, whereas in case of confluent growth, the samples were processed and re-inoculated on fresh LJ slants for obtaining single colonies. Genomic DNA was extracted from each of the single colony isolates using standard protocol. DNA was purified as per manufacturer’s instructions by the QIAGEN column and the DNA preparation kit from illumina. After library preparation, the genomic DNA was fragmented in the range of 100 to 800 bases. The resulting fragmented DNA was cleaned up using QIAquick columns (QIAGEN). The size distribution was checked by running aliquots of the samples on AgilentBioanalyzer 7500 Nano chips. Illumina adapters were ligated to each fragment. Fragments of ~ 300 bases were separated using Gel electrophoresis and sequenced at both ends using illumina GAII sequencer. Sequencing depth in these strains varied from 50 - 200x with average read length of 72 bases.

#### Pre-processing and mapping of Short-reads with reference genome

Sequencing error increases at the end of each cycle, so trimming of short reads is therefore a vital process that could improve the quality of mapping of short reads to the reference genome and identification of single nucleotide variations [46]. Trimming of the short reads was done depending on the average Phred quality score per base in each strain. The average size of reads after trimming was 60 nucleotides. Non-ATGC character containing reads were also filtered before alignment.

*M. tuberculosis* H37Rv (NC_000962.2) was used as reference genome for the mapping of the short reads. All the short reads in each of the strains were separately mapped with the reference genome using Bowtie version 0.12.7 [47]. To make the alignment more stringent, we used two criteria: (a) maximum of two mismatches were in the seed region of the reads and maximum sum of mismatch quality across alignment is less than 70 (b) disallowing all the reads that map at multiple sites in the reference genome. The reference genome coverage of all the strains ranged from 86 - 94% with minimum read depth of 10. Short reads are also aligned following less stringent criteria allowing reads to map multiple positions, which helped in identification of repeat genes.

#### Identification of major and minor SNVs

SNVs were identified from the alignment by using our in-house Perl scripts and were classified into two classes, major SNVs and minor SNVs. Characteristic of major SNVs are: (a) consensus base ratio (*i.e.* number of nucleotides other than the reference divided by total number of nucleotides at a position) more than 0.9, (b) minimum and maximum read depth are 10 and 3 x (average read depth) respectively, (c) consensus base should be supported by reads aligning in both forward and reverse directions, and (d) absence of any other variations present in a 3 bp window. Similarly minor SNVs have: (a) consensus base ratio less than 0.9, (b) more than 10% of the reads in a positions that show the same nucleotide change while the rest of the short reads have the same nucleotide as the reference and (c) variable base should be supported by at least one overlapping paired end reads. Detailed results are listed in Additional file 2: Table S2.

#### Genetic heterogeneity model using simulated short reads

Normally bacterial genome sequencing is carried out using cells derived from a single colony. If the culture is pure, it is expected that all the members of the population will display identical variations as compared to the reference genome with consensus base ratio near 1. On the other hand if the population contains more than one type of bacteria, then some members will show variations while rest will be identical to the reference sequence. These are rare variations. Relative level of a rare variant depends on the nature of the population that is the level of mixing of different genotypes. Here we tried to model the major and minor variations in a population using short read data generated by simulation.

**Figure 9:**
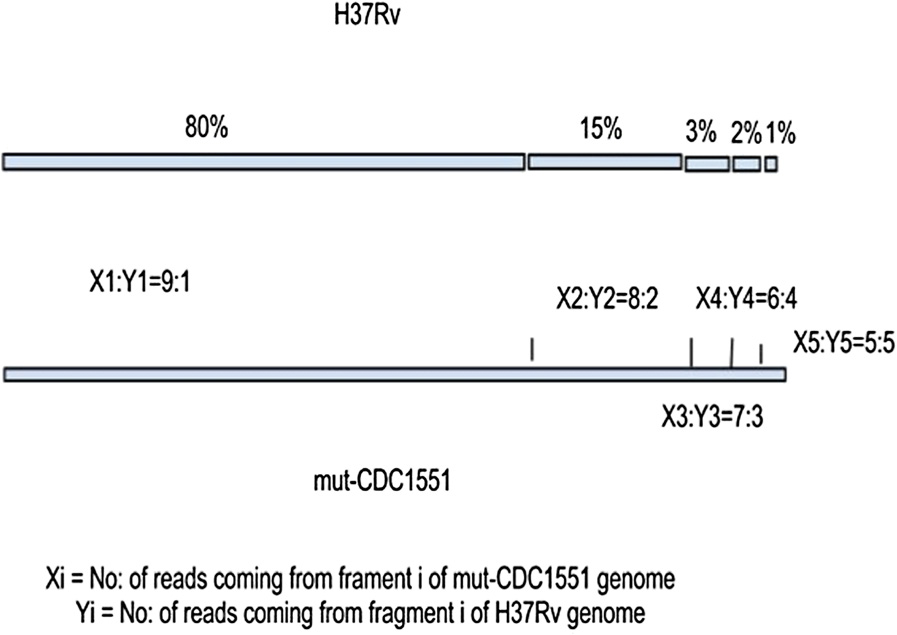
**Cartoon representation showing partitioning of the *M. tuberculosis *H37Rv and CDC1551 genomes used in generating simulated reads.**

At first, *M. tuberculosis* CDC1551 genome was randomly mutated to produce 1200 SNVs and then 10 million paired-end reads were generated using MAQ [48]. *M. tuberculosis* H37Rv was selected as the other genome and partitioned into 5 non-overlapping parts in the proportion 80:15:3:2:1. Paired-end short reads were generated from these five parts with different compositions (See Figure 9).

Let the number of reads generated from CDC1551 be “m”. So, 80% of the genome will have approximately 0.8 x m reads. Now if (0.8 x m)/9 reads from 80% of H37Rv are mixed with these, 80% of tuberculosis genome will be represented by reads from CDC1551 and H37Rv in 9:1 ratio. Similarly 15, 3, 2 and 1% of the genome were represented by 8:2,7:3,6:4 and 5:5 reads from CDC1551 and H37Rv respectively. And then all the reads were aligned with the reference *M. tuberculosis* CDC1551 by bowtie 0.12.7 [47]. After the alignment, randomly generated SNVs would show up as major variations while SNVs unique to H37Rv as rare variants.

The distribution of consensus base ratio in extra-pulmonary isolates and that in simulated data are plotted together (See Figure 5).

#### Identification of missing genes and insertion elements

Missing genes were identified from the alignment of reads with multiple maps from each isolate by identifying regions with minimum read depth below 10 and gene coverage less than 10. These regions are either missed while sequencing or are deleted in the genome of one of the isolates. Large deletions from the isolates were identified using Pindel [17]. As sequencing depth normally achieved is around 100 for the whole genome, there is less chance of missing these regions during sequencing.

**Figure 10:**
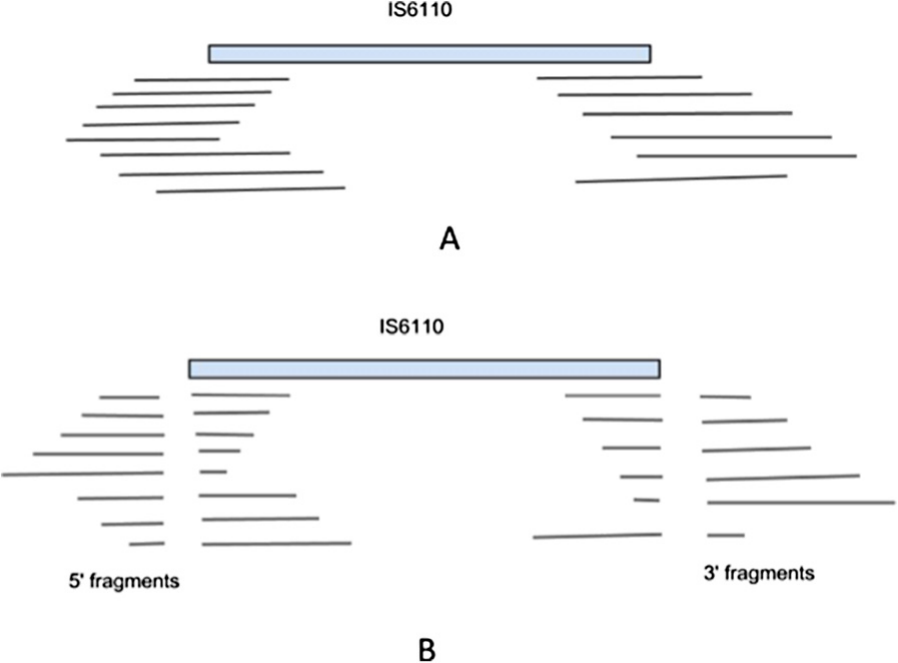
**Cartoon representation of reads aligned to the 5’ and 3’ ends of IS6110.** (**A**) Read aligned to 5’ and 3’ of IS6110 as expected. (**B**) BLAT alignment of fragmented reads in the ends.

Alignment of short reads with multiple matches helped to identify IS elements. The distribution of IS elements in particular IS6110 was done using the following steps. Initially, all reads were aligned against a single 1355 bp copy of IS6110. Alignment was performed using BLAT [49]. Partially aligned or fragmented reads at the 5’ and 3’ ends of the IS elements were then identified (Figure 10) and both 5’ and 3’ fragments were clustered separately using BLASTCLUST (BLAST score-based single-linkage clustering) as reads coming from one genomic region should fall into one cluster. One representative sequence was chosen from each cluster. Let, m is the number of clusters generated from 5’ fragments and n from 3’ fragments. As we do not know specific pairing of 5’ and 3’clusters, m x n sequences were generated by concatenating each 5’ cluster representative with all of the 3’ clusters separately. These m x n sequences were again aligned using BLAT [35] with *M. tuberculosis* H37Rv genome. A near perfect alignment suggested insertion of IS6110 in the region of alignment whereas alignment with around 1355 bp deletion suggested presence of IS6110 in the same position as *H37Rv*.

#### Phylogenetic analysis

Total number of major SNVs identified in all five isolates was 4550 and out of these, 2989 are present in coding regions and only in one of the isolates (unique SNV). We used all unique SNV positions for calculating distance between the isolates using pair wise alignment. Distance based approach (“Neighbor-Joining”) was used to generate phylogenetic relationship (see Figure 8). Branch length indicates divergence distance.

#### Validation of IS element prediction

DNA extraction from the isolates was carried out as described before. Briefly, a single colony of *M. tuberculosis* was picked and suspended in 100 μl of 0.1% TritonX-100. The suspension was boiled in a dry bath at 90°C for 45 min and centrifuged at 10,000 rpm for 10 min. The supernate was used as template DNA in PCRs.

Primers were designed targeting the regions flanking the predicted co-ordinates of the region wherein IS6110 has been predicted to be present. The insertion of IS element would yield a larger PCR amplicon, compared with its absence at the targeted site. The primers used at various co-ordinates with the standardized PCR parameters have been described in Table 3. Briefly amplifications using primer panels were carried out for 35 cycles with 5 min initial and 1 min cyclic denaturation at 95°C; 45 sec annealing at standardized temperature and 2 min cyclic and 7 min final extension at 72°C. For PCR amplification using IS6110 internal primers (Panel H), 30 cycles of denaturation at 95°C for 30 sec, annealing at 65°C for 30 sec and extension at 72°C for 45 sec was carried out. PCRs were set up with reagents obtained from Fermentas AB, Vilnius, Lithuania, using a thermocycler (Applied Biosystems, USA). The amplicons were analyzed on 1.5% agarose gel against a 100 bp DNA Ladder (Thermo Scientific).

#### Spoligotyping of *M. tuberculosis*

Spoligotyping kit was purchased from Isogen Life Science (De Meern, Netherlands). The kit contained the hybridized membrane for spoligotyping, mini-blotter and biotin-labeled primers DRa (5’-GGT TTT GGG TCT GAC GAC-3’) and DRb (5’-CCG AGA GGG GAC GGA AAC-3’) and controls. Primer DRa was biotin labeled hence the amplified PCR product was biotinylated. The detection was done with the streptavidin-POD (peroxidase)-conjugate and chemiluminescence (ECL) detection system. This system was purchased from Roche Applied Science (Mannheim, Germany). Whole procedure was performed according to the manufacturer’s instructions. Detailed procedures were described previously by Kamerbeek et al. 1997. The DNA of different strains was amplified by primers DRa and DRb and the amplified products were hybridized with membranes containing the oligonucleotide probes.

#### PCR reaction

Forward (DRa) and reverse (DRb) primers, dNTP, 10X buffer, Taq and DNA template were mixed together and added to 50 μl double-distilled water. PCR reaction was performed using Taq polymerase under the recommended conditions namely 96°C for 3 min, then at 96°C for 1 min, 55°C for 1 min and 72°C for 1 min. This procedure was followed for 30 cycles and final extension was for 10 min at 72°C.

#### Membrane hybridization

The biotin labeled PCR product was loaded to a mini-blotter for hybridization with the membrane containing the oligo-nucleotide probes. Mini-blotter setup was incubated at 60°C for 60 min. The membrane was then washed at 60°C with 2X SSPE/0.5% SDS for 10 min, followed by incubation with 2X SSPE/0.5% SDS containing 2.5 μl streptavidin-biotin at 42°C for 60 min. Finally, the membrane was washed twice with 2X SSPE/0.5%SDS at 42°C for 60 min and twice with 2X SSPE for 5 min.

#### Detection of hybrid DNA with ECL

The membrane was incubated with the ECL detection system for 1 min and then covered with a transparent plastic film. The membrane was then placed a cassette and exposed to X-ray film.

## Competing interests

The authors declare no competing financial interests.

## Authors’ contributions

AB, HKP and SD conceptualized the study and designed the experiments. AKu and PKu have isolated the genomic DNAs. SD and TR have done the computational analysis. PKa has done wet lab validations. JS and SS performed spoligotyping of the isolates. AB, HKP, SD and TR have written the manuscript. All authors read the manuscript.

## Supplementary Material

Additional file 1 **List of missing genes in each of the isolates.** Row color represent common genes between the isolates. Click here for file

Additional file 2 List of SNVs identified in all the five isolates. Click here for file

Additional file 3 List of synonymous mutation and Relative Synonymous Codon Usage(RSCU). Click here for file

Additional file 4 **The list of SNVs present in all five isolates within the Regulatory regions of genes.** (Regulatory motifs were computationally predicted and stored in MycoRRdb database). Click here for file

Additional file 5 David analysis of isolates ac544. Click here for file

Additional file 6 David analysis of isolates ac74. Click here for file

Additional file 7 David analysis of isolates F85. Click here for file

Additional file 8 David analysis of isolates F99. Click here for file

Additional file 9 David analysis of isolates F85. Click here for file

Additional file 10 Study of IS6110 in different isolates. Click here for file
